# For Toothache

**Published:** 1893-06

**Authors:** 


FOR TOOTHACHE.—In a "popular scientific manual" with the imprint "Trinity College, London," in which only such directions will be given as can be safely carried out "by a laity necessarily ignorant of medical science" are given the following as "some of the many 'infallible' (?) remedies for toothache":

1. Creosote on cotton wool placed in the hollow of the tooth, and covered by cotton wool in mastic varnish.
2. Washing out the mouth with hot water containing bi-carbonate of soda in solution.
3. Insertion of a piece of gall nut into hollow of tooth.
4. Two parts of powdered alum, with seven parts of spirt of nitrous ether applied to the gum and tooth.
5. Warm salt water.
6. The preparations of opium, especially landanum.
7. Alcohol.
8. Tobacco.
9. Cold.
10. Heat.

